# Supplementary material for: Extracorporeal Membrane Oxygenation for COVID 2019-Acute Respiratory Distress Syndrome: Comparison between First and Second Waves (Stage 2)
Source: J Clin Med. 2021 Oct 21;10(21):4839. doi: 10.3390/jcm10214839 (PMC8584595; doi:10.3390/jcm10214839)
Supplement: Supplementary file 1 [file jcm-10-04839-s001.zip › jcm-1392899-supplementary_TD.pdf]

Journal of Clinical Medicine

MDPI

Supplementary Table S1. Biological parameters before ECMO.

Biological parameters before ECMO	All patients (=50)	First Wave (=24)	Second Wave (=26)	P-value
pH	7.4 (7.3-7.4)	7.4 (7.3-7.4)	7.4 (7.3-7.4)	0.327
PaO2/FiO2, mmHg	70 (62-79)	73 (65-84)	68 (57-75)	**0.04**
PaCO2, mmHg	54 (46-63)	52 (42-62)	56 (49-66)	0.067
Bicarbonates, mmol/L	29 (25-36)	29 (23-31)	31 (29-39)	**0.009**
Lactate, mmol/L	1.5 (1.1-1.9)	1.3 (1.1-1.7)	1.6 (1.1-1.9)	0.252
Urea, g/L	0.60 (0.40-0.93)	0.70 (0.30-0.98)	0.60 (0.40-0.93)	0.494
Creatinine, mg/L	8 (6-21)	10 (7-32)	6 (6-17)	0.082
Bilirubin, mg/L	6 (4-10)	6 (5-16)	6 (4-8)	0.363
WBC, 10^9/L	11 (9-15)	11 (9-14)	11 (10-16)	0.315
Lymphocyte count*, 10^9/L	0.7 (0.5-1.1)	0.7 (0.5-1.2)	0.7 (0.4-1.0)	0.209
Hemoglobin, g/dL	9.7 (8.4-10.0)	9.7 (9.1-11.0)	9.0 (8.1- 10.0)	0.217
Hematocrit, %	30 (26-33)	30 (28-33)	29 (25-33)	0.128
Platelets, 10^9/L	258 (199-351)	280 (243-359)	236 (187-314)	0.085
aPTT, ratio	1.5 (1.3-1.8)	1.5 (1.2-2.0)	1.6 (1.2-1.8)	0.995
PT, %	75 (68-84)	74 (65-81)	78 (70-85)	0.163
D-Dimères‡, µg/mL	3.9 (2.3-6.5)	4.0 (2.3-14.0)	3.5 (2.3-4.0)	0.178
Fibrinogen, g/L	7.6 (6.6-8.7)	8.0 (7.2-9.3)	7.1 (5.9-8.1)	**0.009**
CRP, mg/L	250 (107-330)	280 (138-336)	149 (89-313)	0.081
PCT, ng/mL	1.1 (0.4-3.3)	1.8 (0.6-7.1)	0.5 (0.2-1.6)	**0.016**
Ferritine#, ng/mL	1328 (789-2436)	1716 (875-3048)	1281 (638-1893)	0.349

Values are number (%) or median (interquartile range). aPTT : activated partial thromboplastin time.

†1 missing value in first wave group. *1 missing value in first wave group. ‡1 missing value in first wave group and 1 missing value in second wave group. #3 missing values in first wave group and 1 missing value in second wave group.

Supplementary Table S2. ECMO, ventilation, biological parameters and SOFA score at Veno-venous Extracorporeal Membrane Oxygenation (V-V ECMO) Day 3 in First and Second Wave Groups.

Day 3 Characteristics	Parameters	All patients (=49)	First Wave Group (=23)	Second Wave Group (=26)	P-value
ECMO parameters	FmO2 (%)$^{\alpha}$	83 (70-100)	88 (70-100)	80 (68-100)	0.615
	RPM$^{\beta}$	3800 (3325-4041)	3800 (3225-4350)	3750 (3350-4000)	0.513
	ECMO blood flow (L/min)$^{\gamma}$	5.4 (4.7-6)	5.9 (5-6.2)	5.1 (4.6-5.6)	**0.012**
	Sweep gaz flow(L/min)$^{\delta}$	6 (5-7)	6 (5-7.3)	6 (4.9-7)	0.562
	ASV	14	10	4	
	APRV	21	5	16	
	PSV	11	7	4	
	SB	2	0	2	
	FiO2 (%)$^{\varphi}$	50 (40-70)	50 (40-70)	50 (40-80)	0.151
	Vt (mL)$^{\chi}$	230 (180-340)	280 (210-355)	205 (153-310)	0.058
	Vt IBW (mL/kg)$^{\varepsilon}$	3.4 (2.7-5)	4.1 (3.2-5.3)	3.0 (2.4-4.6)	0.074

	RR (cpm)†	20 (18-23)	20 (15-23)	20 (19-24)	0.405
	Pplat (cmH2O)#	24 (21-26)	25 (22-28)	24 (20-26)	0.083
	PEP (cmH2O)‡	12 (10-15)	14 (10-17)	12 (10-12)	0.096
	Driving Pressure (cmH2O)¶	12 (9-14)	12 (8-14)	12 (10-14)	0.709
	Compliance RS (mL/cm H2O)¥	20 (13-32)	23 (20-40)	14 (11-29)	**0.026**
	Mechanical Power (J/min)¤	11.0 (7,1-14.0)	13.0 (9,7-19.0)	8,4 (5,4-12.0)	**0.001**
**Biological parameters**	pH	7.4 (7.4-7.5)	7.4 (7.4-7.5)	7.4 (7.4-7.5)	0.85
	PaO2 (mmHg)	76 (66-88)	77 (70-89)	72 (64-79)	0.133
	PaCO2 (mmHg)	43 (40-48)	41 (38-44)	47 (42-52)	**0.014**
	Bicarbonates (mmol/L)	28 (24-33)	26 (24-29)	31 (27-34)	**0.033**
	Lactate (mmol/L)	1.3 (0.9-2.0)	1.2 (0.9-2.0)	1.3 (0.8-1.8)	0.944
	WBC (10^9/L)	14 (11-18)	15 (11-18)	12 (11-17)	0.394
	Lymphocyte count (10^9/L)[2]	0.8 (0.4-1.3)	0.8 (0.4-1.3)	0.8 (0.4-1.3)	0.686
	Hemoglobin (g/dL)	8.3 (7.7-9.0)	7.9 (6.9-8.8)	8.8 (7.9-9.2)	**0.011**
	Platelets (10^9/L)[ϑ]	173 (106-250)	216 (113-301)	162 (101-201)	0.133
	D-Dimères (µg/mL)[w]	4.0 (3.6-12.0)	3.9 (3.3-10.0)	7.8 (4.0-22.0)	0.145
	Fibrinogen (g/L)	6.2 (4.3-7.4)	6.6 (5.5-8.4)	5.3 (4.2-6.5)	0.117
	aPTT (ratio)	1.7 (1.3-2.5)	1.7 (1.3-2.6)	1.5 (1.3-2.2)	0.367
	Creatinine (mg/L)	9 (6-25)	14 (7-37)	6.5 (5-17)	**0.009**
	Bilirubin (mg/L)	7 (5-17)	17 (6-24)	6 (5-7)	**0.002**
	ASAT (UI/L)	62 (47-132)	73 (52-167)	55 (40-114)	0.058
	ALAT (UI/L)	51 (34-85)	44 (34-79)	54 (33-102)	0.703
	CRP (mg/L)	86 (58-170)	145 (58-191)	83 (52-104)	0.094
	PCT (ng/mL)	0.9 (0.3-2.3)	1.7 (0.7-2.9)	0.4 (0.2-1.2)	**0.021**
**SOFA Day 3**		11 (9-14)	13 (11-15)	10 (8-13)	0.057

Values are number (%) or median (interquartile range). FmO2=fraction of membrane oxygen. RPM=rate per minute. FiO2=fraction of inspired oxygen. ACV=assist-control ventilation. APRV=airway pressure release ventilation. PSV=pressure support ventilation. SB=spontaneous breathing. Vt=Tidal volume. Vt IBW=ideal body weight tidal volume. RR=respiratory rate. Ppeak=Peak pressure. Pplat=plateau pressure. PEEP=positive end-expiratory pressure. Compliance RS = respiratory system compliance. aPTT=activated partial thromboplastin time. ASAT=aspartate aminotransferase. ALAT= alanin aminotransferase.

[α]1 missing value in first wave group, [β]1 missing value in first wave group, [γ]1 missing value in first wave group, [δ]1 missing value in first wave group, ζ2 missing values in first wave group, [φ]1 missing value in first wave group, [χ]1 missing value in first wave group, 2 missing values in second wave group, [ε]2 missing values in second wave group, †1 missing value in first wave group, 2 missing values in second wave group, #1 missing value in first wave group, 2 missing values in second wave group, ‡1 missing value in first wave group, 2 missing values in second wave group, ¶2 missing values in first wave group, 1 missing value in second wave group, ¥1 missing value in first wave group, 2 missing values in second wave group, ¤1 missing value in first wave group, 2 missing values in second wave group, [2]1 missing value in first wave group, [ϑ]1 missing value in first wave group, [w]1 missing value in first wave group, 3 missing values in second wave group.

Supplementary Table S3. ECMO, ventilation, biological parameters and SOFA score at Veno-venous Extracorporeal Membrane Oxygenation (V-V ECMO) Day 7 in First and Second Wave Groups.

Day 7 Characteristics	Parameters	All patients (=41)	First Wave Group (=17)	Second Wave Group (=24)	P-value
**ECMO parameters**	FmO2 (%)	80 (65-100)	80 (70-100)	75 (60-100)	0.693
	RPM	3600 (3100-4038)	4000 (2850-4400)	3500 (3200-3950)	0.233
	ECMO blood flow (L/min)	5.3 (4.5-6)	5.9 (4.5-6.9)	5.1 (4.4-5.5)	**0.048**
	Sweep gaz flow (L/min)	7 (4.5-8)	6 (5-8.5)	7 (4-8)	0.974
	ACV	10	6	4	
	APRV	19	6	13	
	PSV	11	5	6	
	SB	1	0	1	
	FiO2 (%)	50 (40-65)	50 (45-65)	55 (40-68)	0.626
	Vt (mL)$^{\chi}$	225 (153-375)	270 (210-395)	180 (130-360)	**0.045**
	Vt IBW (mL/kg)$^{\varepsilon}$	3.4 (2.3-5.1)	4.0 (3.3-5.6)	2.7 (1.9-5.1)	**0.02**
	RR (cpm)†	22 (17-28)	22 (17-28)	24 (15-26)	0.844
	Pplat (cmH2O)#	25 (24-29)	28 (25-30)	24 (23-26)	**0.031**
	PEEP (cmH2O)‡	12 (10-14)	12 (10-17)	10 (10-12)	0.134
	Driving Pressure (cmH2O)¶	14 (11-15)	14 (11-16)	14 (11-15)	0.551
	Compliance RS (mL/cm H2O)¥	18 (10-28)	20 (15-28)	16 (9-28)	0.165
	Mechanical Power (J/min)¤	11 (7-25)	19 (9-30)	7 (6-22)	**0.02**
**Biological parameters**	pH	7.4 (7.4-7.5)	7.4 (7.4-7.4)	7.4 (7.4-7.5)	0.552
	PaO2 (mmHg)	70 (61-85)	78 (73-94)	65 (58-75)	**0.005**
	PaCO2 (mmHg)	44 (40-50)	41 (39-47)	45 (41-52)	0.112
	Bicarbonates (mmol/L)	29 (24-31)	25 (23-31)	30 (27-32)	0.064
	Lactate (mmol/L)	1.2 (0.1-1.6)	1.2 (0.8-1.6)	1.2 (1.1-1.6)	0.338
	WBC (10^9/L)	15 (11-21)	18 (14-21)	12 (9-18)	**0.014**
	Lymphocyte count (10^9/L)	0.9 (0.5-1.6)	1.5 (1.0-2.0)	0.8 (0.4-1.1)	**0.001**
	Hemoglobin (g/dL)	8.3 (7.6-8.8)	7.9 (7.3-8.3)	8.5 (7.7-9.0)	0.075
	Platelets (10^9/L)	112 (75-169)	135 (89-185)	92 (64-141)	**0.05**
	D-Dimères (µg/mL)^{w}	4.0 (3.7-16.0)	8.1 (3.3-16.0)	4.0 (3.9-29.0)	0.798
	Fibrinogen (g/L)	4.4 (2.8-6.0)	6.0 (3.8-7.9)	4.0 (2.7-5.2)	**0.005**
	aPTT (ratio)	1.7 (1.3-2.4)	1.5 (1.3-2.1)	1.8 (1.2-2.6)	0.382
	Creatinine (mg/L)	9 (5-17)	16 (8-25)	7 (4-14)	**0.007**
	Bilirubin (mg/L)	7 (5-14)	9 (6-45)	6 (5-7)	**0.007**
	ASAT (UI/L)	49 (41-87)	61 (40-129)	48 (42-70)	0.239
	ALAT (UI/L)	46 (31-72)	42 (30-63)	49 (34-84)	0.209

	CRP (mg/L)	85 (32-220)	153 (22-306)	78 (33-189)	0.56
	PCT (ng/mL)	0.6 (0.2-2.5)	1,3 (0.6-7.9)	0.4 (0.2-1.2)	**0.018**
**SOFA Day 7**		11 (8-16)	14 (9-18)	10 (8-14)	0.201

Values are number (%) or median (interquartile range). FmO2=fraction of membrane oxygen. RPM=rate per minute. FiO2=fraction of inspired oxygen. ACV=assist-control ventilation. APRV=airway pressure release ventilation. PSV=pressure support ventilation. SB=spontaneous breathing. Vt=Tidal volume. Vt IBW=ideal body weight tidal volume. RR=respiratory rate. Ppeak=Peak pressure. Pplat=plateau pressure. PEEP=positive end-expiratory pressure. Compliance RS = respiratory system compliance. aPTT=activated partial thromboplastin time. ASAT=aspartate aminotransferase. ALAT= alanin aminotransferase.

[χ]1 missing value in second wave group, [ε]1 missing value in second wave group, †1 missing value in second wave group, #1 missing value in second wave group, ‡1 missing value in second wave group, ¶1 missing value in second wave group, ¥1 missing value in second wave group, ¤1 missing value in second wave group, [w]4 missing values in second wave group.

Supplementary Table S4. Treatments under ECMO

Treatments under ECMO	All patients (=50)	First Wave (=24)	Second Wave (=26)	P-value
**Adjuvant ARDS treatment**				
Prone Positioning	34 (68)	13 (54.2)	21 (80.8)	**0.044**
Inhaled nitric oxide	33 (66)	13 (54.2)	20 (76.9)	0.09
Almitrine	14 (28)	5 (20.8)	9 (34.6)	0.278
**COVID-19 therapies**				
Glucocorticoids	44/50 (88)	22/24 (91.7)	22/26 (84.6)	0.669
Antiviral	1 (2)	1 (4.2)	0 (0)	0.48
Immunomodulators	2/50 (4)	2/24 (8.3)	0/26 (0)	0.225
**Renal replacement therapy**	22 (44)	15 (62.5)	7 (26.9)	**0.011**
**Blood-product transfusion**				
Red cells transfusion	49 (98)	24 (100)	25 (96.2)	1
Platelets transfusion	17 (34)	6 (25)	11 (42.3)	0.197
Fresh frozen plasma	14 (28)	5 (20.8)	9 (34.6)	0.278
Fibrinogen concentrate	7 (14)	2 (8.3)	5 (19.2)	0.42

Values are number (%). Antiviral therapies were Lopinavir-Ritonavir, Chloroquine, Remdesivir. Immunomodulators were intravenous immunoglobulin, anti-cytokine, JAK inhibitors.
